# HIV Impairs Opsonic Phagocytic Clearance of Pregnancy-Associated Malaria Parasites

**DOI:** 10.1371/journal.pmed.0040181

**Published:** 2007-05-29

**Authors:** Jessica Keen, Lena Serghides, Kodjo Ayi, Samir N Patel, John Ayisi, Anne van Eijk, Richard Steketee, Venkatachalam Udhayakumar, Kevin C Kain

**Affiliations:** 1 Faculty of Medicine, University of Toronto, Toronto, Canada; 2 McLaughlin-Rotman Centre, McLaughlin Center for Molecular Medicine, University of Toronto, Toronto, Canada; 3 University Health Network, Toronto, Canada; 4 Center for Vector Biology and Control Research, Kenya Medical Research Institute, Kisumu, Kenya; 5 Division of Parasitic Diseases, Centers for Disease Control and Prevention, Atlanta, Georgia, United States of America; Seattle Biomedical Research Institute, United States of America

## Abstract

**Background:**

Primigravid (PG) women are at risk for pregnancy-associated malaria (PAM). Multigravid (MG) women acquire protection against PAM; however, HIV infection impairs this protective response. Protection against PAM is associated with the production of IgG specific for variant surface antigens (VSA-PAM) expressed by chondroitin sulfate A (CSA)-adhering parasitized erythrocytes (PEs). We hypothesized that VSA-PAM-specific IgG confers protection by promoting opsonic phagocytosis of PAM isolates and that HIV infection impairs this response.

**Methods and Findings:**

We assessed the ability of VSA-PAM-specific IgG to promote opsonic phagocytosis of CSA-adhering PEs and the impact of HIV infection on this process. Opsonic phagocytosis assays were performed using the CSA-adherent parasite line CS2 and human and murine macrophages. CS2 PEs were opsonized with plasma or purified IgG subclasses from HIV-negative or HIV-infected PG and MG Kenyan women or sympatric men. Levels of IgG subclasses specific for VSA-PAM were compared in HIV-negative and HIV-infected women by flow cytometry. Plasma from HIV-negative MG women, but not PG women or men, promoted the opsonic phagocytosis of CSA-binding PEs (*p* < 0.001). This function depended on VSA-PAM-specific plasma IgG1 and IgG3. HIV-infected MG women had significantly lower plasma opsonizing activity (median phagocytic index 46 [interquartile range (IQR) 18–195] versus 251 [IQR 93–397], *p* = 0.006) and levels of VSA-PAM-specific IgG1 (mean fluorescence intensity [MFI] 13 [IQR 11–20] versus 30 [IQR 23–41], *p* < 0.001) and IgG3 (MFI 17 [IQR 14–23] versus 28 [IQR 23–37], *p* < 0.001) than their HIV-negative MG counterparts.

**Conclusions:**

Opsonic phagocytosis may represent a novel correlate of protection against PAM. HIV infection may increase the susceptibility of multigravid women to PAM by impairing this clearance mechanism.

## Abstract 2

Based on a comparison of HIV-negative or HIV-infected primigravid and multigravid women, Kevin Kain and colleagues suggest that opsonic phagocytosis might protect against pregnancy-associated malaria, and that HIV infection might impair this parasite clearance mechanism.

## Editors' Summary

**Background.:**

Every year, malaria kills more than one million people—mostly young children. Among adults, pregnant women are most affected by malaria, a parasitic disease spread by mosquitos. In areas of Africa where malaria is widespread, about 10,000 women die because of pregnancy-associated malaria (PAM) each year. In PAM, red blood cells containing parasites (parasitized erythrocytes or PEs) collect in the woman's placenta. These PEs, which stick to a placental molecule called chondroitin sulfate A (CSA), are covered with parasitic proteins known as variant surface antigens of PAM (VSA-PAM). Women in their first pregnancy (primigravid women) are particularly susceptible to PAM, but multigravid women are more resistant unless they are also infected with HIV (the virus that causes AIDS), in which case they are extremely susceptible to PAM. Protection against PAM in multigravid women is associated with the production of immunoglobulins (proteins made by the immune system that circulate in the blood and bind to foreign proteins or antigens) that recognize VSA-PAM. These immunoglobulins or antibodies are called VSA-PAM-specific IgG and their production increases with each pregnancy

**Why Was This Study Done?:**

It is unclear how VSA-PAM-specific IgG protects multigravid women against PAM or how HIV infection impairs this protective response. One possibility is that VSA-PAM-specific IgG coats the PEs in the placenta to enable immune system cells called macrophages to recognize and ingest them, a process called opsonic phagocytosis. In this study, the researchers have investigated whether opsonic phagocytosis provides multigravid women with protection against PAM and whether a failure of this form of protective immunity underlies the susceptibility of HIV-infected multigravid women to PAM.

**What Did the Researchers Do and Find?:**

The researchers collected plasma (the fluid part of blood) from primigravid and multigravid women (some of whom were infected with HIV) living in a Kenyan region where malaria is common soon after they gave birth and from men living in the same area. They then purified IgG1, IgG2, IgG3, and IgG4 from the plasma samples. These four IgG subclasses have different immune functions—only IgG1 and IgG3 participate in opsonic phagocytosis. To measure the ability of the plasma samples and purified IgGs to promote opsonic phagocytosis, the researchers mixed each sample with a laboratory isolate of CSA-binding PEs and macrophages and counted how many PEs the macrophages ingested. Plasma from HIV-negative multigravid women but not primigravid women or men promoted opsonic phagocytosis and, in the plasma from multigravid women, this activity depended on IgG1 and IgG3. HIV-infected multigravid women, however, had less opsonizing activity in their plasma than HIV-negative multigravid women and the level of this activity correlated with the levels of VSA-PAM-specific IgG1 and IgG3 (measured using a technique called flow cytometry) in the plasma samples of these two groups of women.

**What Do These Findings Mean?:**

These findings support the hypothesis that VSA-PAM-specific IgG1 and IgG3 promote opsonic clearance of CSA-binding PEs. Thus, opsonic immune mechanisms may be involved in the protective response to PAM seen in multigravid women. However, because all the measurements of opsonic phagocytosis in this study used a laboratory isolate of PEs, these findings need to be confirmed using PEs isolated from placentas to check that they are generalizable. Other findings reported here suggest that HIV-positive multigravid women are more susceptible to PAM than HIV-negative multigravid women because reduced amounts of VSA-PAM-specific IgG in their plasma reduce the ability of opsonic phagocytosis to clear PEs from their placenta. Overall, these results may have implications for the development of vaccines against PAM. For example, they suggest that vaccines should be designed to stimulate the production of VSA-PAM-specific antibodies of the IgG1 and IgG3 subclasses. They also suggest that, provided the link between opsonic phagocytosis and protection against PAM can be confirmed in population-based studies, new vaccines could be evaluated for their potential to protect women against PAM by seeing whether the plasma of vaccinees promotes opsonic phagocytosis of CSA-binding PEs.

**Additional Information.:**

Please access these Web sites via the online version of this summary at http://dx.doi.org/10.1371/journal.pmed.0040181.

- US Centers for Disease Control and Prevention information on malaria and malaria during pregnancy (in English and Spanish)
- World Health Organization information on malaria, including a feature on malaria in pregnancy (in English, Spanish, French, Arabic, Chinese and Russian)
- Special Program for Research and Training in Tropical Diseases (TDR) information on malaria and research into the disease
- Roll Back Malaria Partnership fact sheets on all aspects of malaria, including malaria in pregnancy (in English, French and Portuguese)
- HIV Insite, information from the University of California at San Francisco on malaria and HIV

## Introduction

In malaria-endemic areas, pregnant women are more likely to acquire malaria and to have higher parasite burdens than nonpregnant individuals [1]. Pregnancy-associated malaria (PAM), especially in primigravidae, has profound maternal and fetal health consequences including maternal anaemia and delivery of low birth-weight infants [2,3]. The effects of HIV infection on maternal health are superimposed on those of malaria in sub-Saharan Africa, where approximately 1 million pregnancies each year are complicated by malaria-HIV coinfection [4]. HIV infection in pregnancy is associated with higher rates of clinical malaria, higher parasite densities (both peripheral and placental), and a higher risk of maternal anaemia and low birth-weight infants [5]. While HIV infection affects all gravidities, the HIV-associated risk of malaria is consistently greater in multigravidae [5].

Sequestration of parasitized erythrocytes (PEs) within the placenta is thought to be the fundamental pathological mechanism underlying PAM. The placenta appears to select for a subpopulation of PEs (PAM PEs) that express novel variant surface antigens (VSA-PAM) and adhere to chondroitin sulfate A (CSA), a glycosaminoglycan found in the placenta [6,7]. The Plasmodium falciparum erythrocyte membrane protein 1 (PfEMP1) family has been identified as the major VSA mediating adhesion to CSA [8,9]. PfEMP1 proteins are encoded by *var* genes and are expressed on the PE surface in a mutually exclusive manner [10]. Recently *var2csa* was shown to be the principal *var* gene transcribed in placental isolates [11] that encodes CSA-binding PfEMP1 [12,13].

Protection against PAM and its clinical consequences correlates with the acquisition of VSA-PAM-specific IgG in a parity-dependent manner [14–17]. Levels of VSA-PAM-specific antibodies are low to nonexistent in primigravid (PG) women and nonpregnant individuals, and tend to increase in multigravid (MG) women with increasing gravidity [14,16,17]. The presence of HIV infection has been associated with reduced levels of VSA-PAM-specific IgG, suggesting a potential mechanism by which HIV may impair the acquisition or maintenance of immunity to PAM in multigravidae [18].

VSA-PAM-specific IgG is thought to protect against PAM by blocking the adhesion of PEs to CSA in the placenta [15,16,19]. However, some studies have failed to demonstrate an association between adhesion-blocking antibodies and protection against PAM [18,20,21]. Furthermore, plasma levels of VSA-PAM-specific IgG do not always correlate with the ability of the same plasma to block adhesion of PEs to CSA [18,20,21]. Taken together, these data suggest that in addition to adhesion blocking, VSA-PAM-specific IgG may mediate protection against PAM through other immunologic mechanisms.

Recently, the IgG subclass profile of malaria-exposed pregnant women has been investigated. IgG1 and IgG3 are the classic cytophilic IgG subclasses, whereas IgG2 and IgG4 are typically unable to promote opsonic phagocytosis because of their weak affinity for Fcγ receptors. The IgG subclass profile of pregnant women toward VSA-PAM was found to be dominated by IgG1 and IgG3 subclasses [22–24], suggesting that opsonic phagocytosis might be an important determinant in the control of placental parasitaemia. The purpose of this study was to investigate IgG-mediated mechanisms of protection against PAM and the impact of HIV infection on this response. We hypothesized that (1) VSA-PAM-specific IgG may contribute to protection against PAM by promoting the opsonic phagocytosis of CSA-binding PEs and (2) HIV infection results in the inability to generate or sustain a cytophilic antibody response to PAM PEs, rendering the HIV-infected MG woman susceptible to PAM. We show that CSA-binding PEs are cleared by opsonic phagocytosis in a sex-specific and parity-dependent manner. This effector function was mediated by VSA-PAM-specific IgG1 and IgG3, but not IgG2 or IgG4. We further demonstrate that plasma from HIV-infected MG women had significantly lower opsonizing activity than HIV-negative MG women, and this was associated with lower levels of VSA-PAM-specific IgG1 and IgG3.

## Methods

### Study Population

Plasma samples were obtained at one month postpartum from PG and MG (parity 3–5) women, with and without HIV infection, living in an area of holoendemic malaria transmission in Western Kenya. These women were participants in a cohort study to assess the impact of placental malaria infection on mother-to-child transmission of HIV infection [25]. Plasma samples used in this study were randomly selected from this cohort. Of the 38 HIV-infected women studied, 29 (76%) had CD4^+^ T cell counts above 400 cells/μl, 8 (21%) had counts between 200–400 cells/μl, and one patient had a CD4^+^ T cell count below 200 cells/μl. Plasma from malaria-exposed men living in the same region of Western Kenya or unexposed Canadian donors was used as controls in each assay. Mean age differed significantly between PG and MG women (mean age in years [standard deviation (SD)] PG 18.6 [1.87] versus MG 25.79 [3.58], *p* < 0.001, by Fisher exact test) but did not differ significantly between MG women and men (MG 25.79 [3.58] versus men 26.17 [4.93], *p* = 0.76, by Fisher exact test). Malaria exposure (urban versus semi-urban), past history of malaria and presence of parasitaemia at time of delivery did not differ significantly between PG and MG women. None of the women included in this study received intermittent preventive treatment or chemoprophylaxis during their pregnancy. This study was approved by the institutional review boards of the Kenya Medical Research Institute and the Centers for Disease Control and Prevention, Atlanta, Georgia, United States. Informed consent was obtained from all participants.

### P. falciparum Culture

The laboratory line CS2 was cultured in vitro as described [26,27]. Cultures were routinely treated with mycoplasma removal agent (ICN), and were tested and found negative for mycoplasma by PCR.

### Plasma IgG Fractionation

Total IgG was purified from other plasma components using a Hitrap Protein G HP Column (Amersham Biosciences, http://www5.amershambiosciences.com). Neat plasma (200 μl) was diluted in 20 mM sodium phosphate buffer (pH 7) and applied to the column. Nonbound plasma proteins were washed through the column with 20 mM sodium phosphate buffer (pH 7) and collected (wash fraction). Bound IgG was eluted from the column using 0.1 M glycine-HCl (pH 2.7) and collected in fractions (eluate fraction) containing 1 M Tris-HCl (pH 9) to preserve the activity of acid-labile IgGs. The presence of protein in wash and eluate fractions was confirmed by a Bradford protein assay. Both fractions were dialyzed against PBS, concentrated to the initial plasma volume using Ultrafree-CL centrifugal filter devices (30,000 kDa) (Millipore, http://www.millipore.com), and stored at −20 °C.

### Plasma Fractionation of Cytophilic and Noncytophilic IgG Subclasses

To purify cytophilic IgG subclasses from noncytophilic IgG subclasses, an affinity matrix with specificity for IgG2 and IgG4 (IgG2+4 affinity matrix) was prepared using the AminoLink Plus Immobilization Kit (Pierce, http://www.piercenet.com) according to the manufacturer's instructions. Purified mouse monoclonal antibodies (anti-IgG2: HP6014, 4 mg; anti-IgG2: HP6002, 4 mg; anti-IgG4: HP-6025, 2 mg) (Hybridoma Reagent Laboratory, http://www.hybridoma-reagent-laboratory.com) were used for matrix preparation.

Neat plasma (200 μl) diluted in 0.1 M phosphate, 0.15 M NaCl (pH 7.2) was incubated with the IgG2+4 affinity matrix for 1 h at room temperature. IgG1 and IgG3 were washed through the matrix with 0.1 M phosphate, 0.15 M NaCl (pH 7.2) and collected (wash fraction). IgG2 and IgG4 were eluted from the matrix using 0.1 M glycine-HCl (pH 2.7) and collected as above (eluate fraction). Both fractions were dialyzed against PBS, concentrated to the initial plasma volume using Amicon Ultra-15 Centrifugal Filter Units (50,000 kDa) (Millipore), and stored at −20 °C.

### Detection of Purified IgG Subclasses by Slot Blot

IgG subclasses were detected in the purified wash and eluate fractions using a slot blot. Membranes were probed with anti-human IgG1 (HP-6001, 1:5,000), anti-human IgG2 (HP-6014, 1:3,000), anti-human IgG3 (HP-6050, 1:3,000), and anti-human IgG4 (HP-6025, 1:10,000) (Sigma-Aldrich, http://www.sigmaaldrich.com) for 1 h at room temperature, washed four times with PBST and incubated with goat anti-mouse HRP secondary (1:4,000) (Bio-Rad, http://www.bio-rad.com) for 1 h at room temperature. IgG subclasses were detected by enhanced chemiluminescence (ECL).

### In Vitro Opsonic Phagocytosis Assays

Phagocytosis assays were performed using primary human monocytes and *Cd36^+/+^* or *Cd36* ^−*/*−^ murine macrophages as previously described [26,28]. Macrophages (2.5 × 10^5^) were plated on glass coverslips and cultured for 3 d prior to use. To opsonize PEs, 50 μl of trophozoite-stage CS2 PEs (5% haematocrit, parasitaemia 4%–10%) in RPMI-1640 were incubated with 25 μl of plasma (1:3 dilution) for 30 min at 37 °C. In some cases, plasma was heat-inactivated for 1 h at 56 °C prior to use. Opsonized PEs were washed and added to macrophages at a ratio of 20 PEs per macrophage and incubated at 37 °C for 2 h. The coverslips were then washed in ice-cold water for 30–40 s, fixed, and stained using Diff-Quick. Phagocytosis was quantified microscopically, with at least 500 cells counted per coverslip, and presented as a phagocytic index, defined as the total number of internalized PEs per total number of macrophages counted expressed as a percentage. All assays were performed and interpreted by staff blinded to the clinical and demographic data. Assays were performed in duplicate or triplicate and repeated at least once.

### Flow Cytometry

Plasma was first preadsorbed with noninfected RBCs for 30 min at room temperature to remove nonspecific antibodies directed against RBC antigens. Trophozoite-stage CS2 PEs (4%–8% parasitaemia) were washed, resuspended in 2% heat-inactivated fetal bovine serum (FBS)/PBS to a haematocrit of approximately 0.1%, and incubated with preadsorbed human plasma (1:10 dilution) in a 96-well plate for 30 min at RT. PEs were then washed three times with 2% FBS/PBS, and incubated with mouse anti-human IgG1 (1:50) (HP6069, Zymed, http://www.invitrogen.com), IgG2 (1:20) (HP6014, Sigma-Aldrich), IgG3 (1:20) (HP6047, Zymed), or IgG4 (1:50) (HP6025, Sigma-Aldrich) diluted in 2% FBS/PBS for 30 min at RT. PEs were washed and incubated for 30 min at RT with Atto-488 goat anti-mouse IgG (1:50) (Sigma-Aldrich) and ethidium bromide (EtBr) (0.5 μg/well) diluted in 2% FBS/PBS. PEs were fixed in 2% paraformaldehyde, and analysed on a Becton Dickinson FACSCalibur flow cytometer. Data were analysed using FlowJo software. 10,000 EtBr-positive events were collected. For each sample, the Atto-488 mean fluorescence intensity (MFI) of the EtBr-positive population was recorded. Plasma from unexposed donors and pooled plasma from HIV-negative MG Kenyan women were used as negative and positive controls, respectively, in each assay. All assays and readings were performed blinded to clinical and demographic data.

### Statistical Analysis

All data were tested for normality using the D'Agostino and Pearson omnibus normality test. If any of the groups being compared failed the normality test, nonparametric tests were used for all comparisons. The Mann-Whitney rank sum test and the Spearman correlation coefficient were used to assess statistically significance. Statistical analyses were performed using SigmaStat (http://www.systat.com) and GraphPad Prism (http://www.graphpad.com).

## Results

### Opsonizing Activity of Maternal Plasma

**Figure 1:**
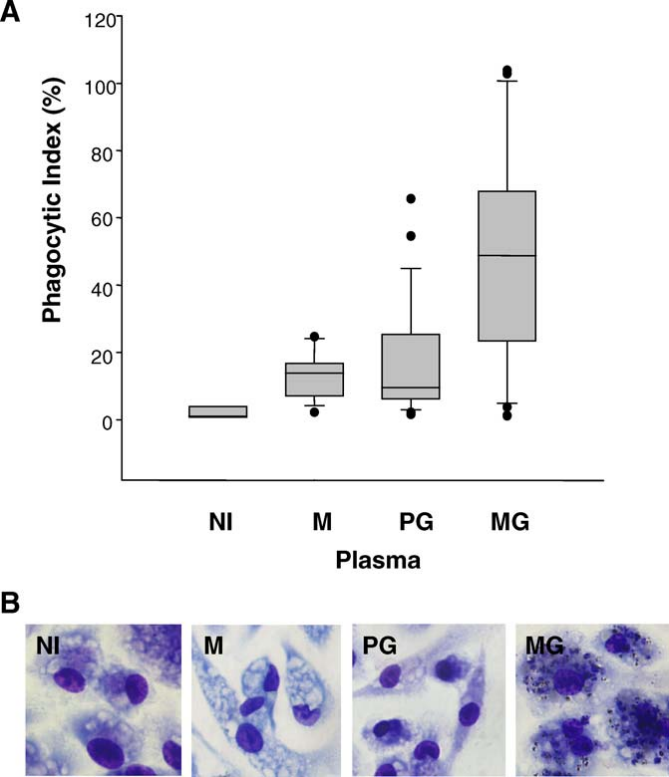
Plasma Opsonizing activity of CSA-Binding Parasitized Erythrocytes Is Sex-Specific and Parity-Dependent (A) Box (median and IQR) and whisker (range) plot showing phagocytic index of CSA-binding (CS2) PEs opsonized with plasma from nonimmunes (NI) (*n* = 3), malaria-exposed males (M) (*n* = 14), malaria-exposed PG (*n* = 21), or malaria-exposed MG (*n* = 16) donors. Statistical significance was assessed by Mann-Whitney rank sum test. *M versus MG *p* = 0.0015; PG versus MG *p* = 0.0031. (B) Photomicrographs show examples of murine macrophage internalization of CSA-binding (CS2) PEs opsonized with plasma from individual donors.

VSA-PAM-specific IgG is thought to protect against PAM by blocking CSA-adhering PEs in the placenta; however, these antibodies may also protect by promoting the opsonic phagocytic clearance of PAM PEs. To test whether opsonic phagocytosis might be an important determinant in the control of placental parasitaemia, we compared the ability of plasma from PG and MG Kenyan women (see Table S1), as well as sympatric men, to promote the opsonic phagocytic clearance of trophozoite-stage CS2 PEs. CS2 expresses *var2csa* as its dominant *var* transcript and displays an antigenic and adhesive phenotype similar to clinical isolates causing placental malaria [13,18]. Opsonic phagocytosis assays were performed in vitro using primary human monocytes as well as *Cd36^+/+^* and *Cd36* ^−*/*−^ murine macrophages. *Cd36* ^−*/*−^ macrophages were used to eliminate the possibility of scavenger receptor-mediated uptake [28]. Results obtained were similar for each macrophage type (see Figure S1), thus only data for *Cd36* ^−*/*−^ macrophages are presented here. As shown in Figure 1, plasma from unexposed donors, as well as from malaria-exposed PG women and males, induced uniformly low opsonic uptake of CSA-binding PEs. Conversely, plasma from MG women induced significantly higher opsonic phagocytosis of CS2 PEs than plasma from malaria-exposed PG women or men (median phagocytic index: MG 49 [interquartile range (IQR) 23–68], PG 9.6 [IQR 6–27], men 14 [IQR 7–19]; MG versus PG, *p =* 0.0031 [U = 71.0]; MG versus men *p* = 0.0015 [U = 35.0], by Mann-Whitney rank sum test). The higher plasma opsonizing activity of MG donors compared with PG and male donors was specific for CS2 PEs and did not appear to be confounded by age, since plasma from all donors similarly promoted the opsonic phagocytic uptake of the non-CSA-binding parasite line ITG (Figure S2). In addition, heat inactivation of complement in plasma from MG women had no effect on plasma opsonizing activity (Figure S3). These results demonstrate that plasma-induced opsonic phagocytosis of CSA-binding PEs is sex-specific and parity-dependent, a pattern that parallels the production of VSA-PAM-specific IgG.

### Role of IgG and IgG Subclasses in Mediating Opsonic Phagocytosis

**Figure 2:**
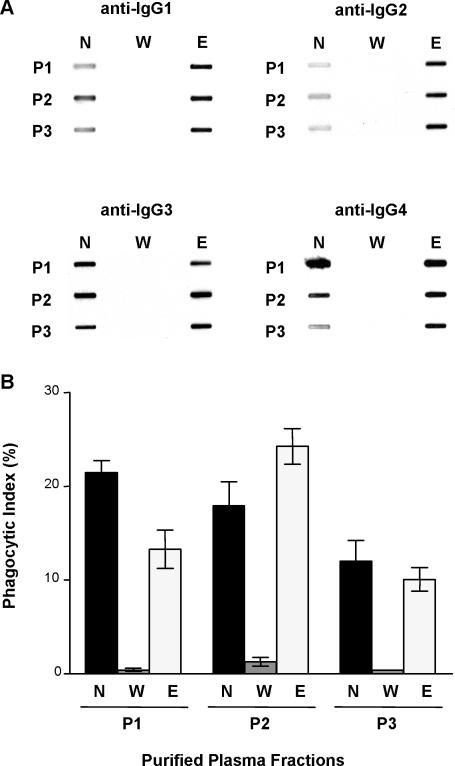
VSA-PAM-Specific IgG Mediates Opsonic Phagocytosis of CSA-Binding Parasitized Erythrocytes (A) Slot blot detection of IgG subclasses in neat plasma (N) and Protein G column-purified wash (W) and eluate (E) plasma fractions. (B) Bar graph showing phagocytic index of plasma opsonized CS2 PEs by murine macrophages. Results are presented as the mean with SD of triplicates (*n* = 3) for three MG patients (P1, P2, and P3).

To confirm that VSA-PAM-specific IgG was the plasma component mediating the opsonic phagocytosis of CS2 PEs, plasma IgG from MG women was purified using a Protein G column. All IgG subclasses bound the column and were detected in the eluate fraction (Figure 2A), while other plasma proteins remained in the wash fraction. As expected, no IgG was detected in the wash fraction (Figure 2A). Opsonic phagocytosis assays were then performed using the purified fractions to opsonize CS2 PEs. As shown in Figure 2B, only the eluate (IgG-containing) fraction induced opsonic phagocytosis, suggesting that VSA-PAM-specific IgG, and not some other plasma component, mediates the opsonic phagocytic clearance of CS2 PEs.

**Figure 3:**
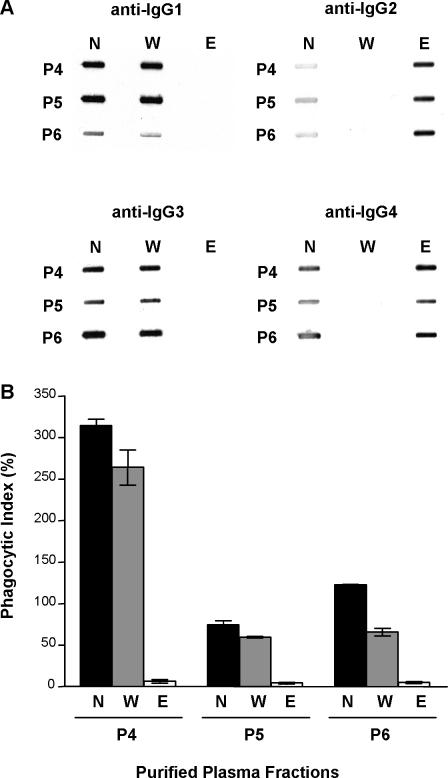
VSA-PAM-Specific IgG1 and IgG3 Promote Opsonic Phagocytosis of CSA-Binding Parasitized Erythrocytes (A) Slot blot detection of IgG subclasses in neat plasma (N) and IgG2+4 affinity matrix-purified wash (W) and eluate (E) plasma fractions. (B) Bar graph showing phagocytic index of plasma opsonized CS2 PEs by murine macrophages. Results are presented as the mean with SD of triplicates (*n* = 3) for three MG patients (P4, P5, and P6).

To assess the relative contribution of the IgG subclasses in mediating opsonic phagocytosis, we separated the cytophilic and noncytophilic IgG subclasses from MG plasma. Figure 3A shows the results of the affinity matrix IgG subclass purification. IgG1 and IgG3 were detected in the wash fraction, whereas IgG2 and IgG4 were detected in the eluate fraction (Figure 3A). CS2 PEs were opsonized with each fraction and their uptake by macrophages was measured. As shown in Figure 3B, only IgG1 and IgG3, but not IgG2 and IgG4, promoted the opsonic phagocytosis of CS2 PEs.

### Influence of HIV on Plasma Opsonizing Activity

**Figure 4:**
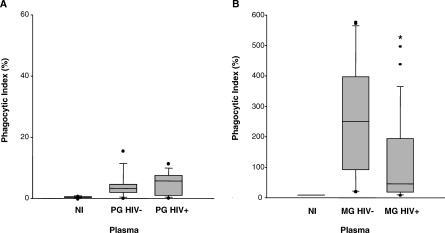
HIV-Infected Multigravid Women Have Impaired Plasma Opsonizing Activity against CSA-Binding Parasitized Erythrocytes Box (median and IQR) and whisker (range) plot showing phagocytic index of CS2 PEs opsonized with plasma from (A) NI (*n* = 6), PG HIV− (*n* = 9), or PG HIV+ (*n* = 11) donors, and (B) NI (*n* = 2), MG HIV− (*n* = 23), or MG HIV+ (*n* = 23) donors. Statistical significance was assessed by Mann-Whitney rank sum test. *MG HIV− versus MG HIV+ *p* = 0.006. MG HIV+, HIV-infected MG; MG HIV−, HIV-negative MG; NI, nonimmune; PG HIV−, HIV-negative PG; PG HIV+, HIV-infected PG.

**Figure 5:**
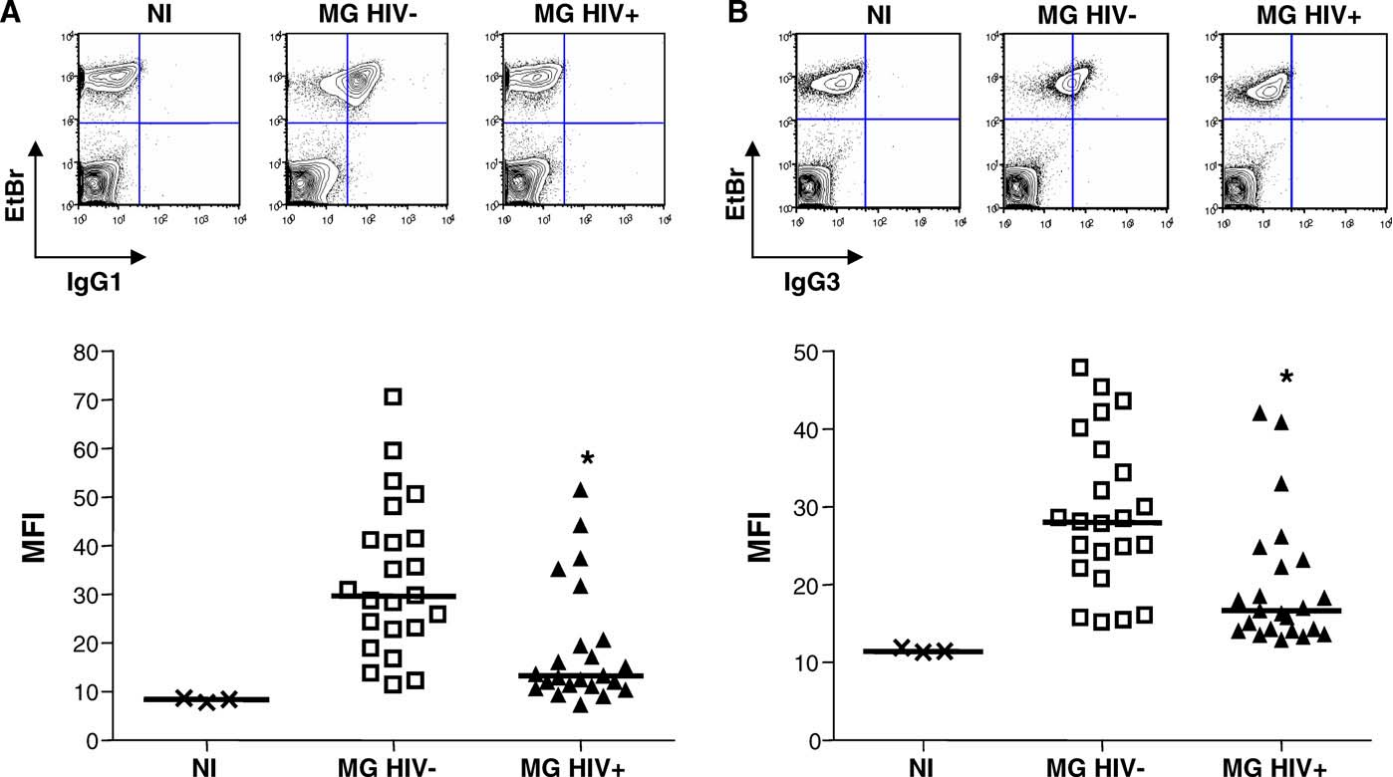
HIV-Infected Multigravid Women Have Lower Levels of VSA-PAM-Specific IgG1 and IgG3 Flow cytometry was used to measure levels of IgG1 (A) and IgG3 (B) with specificity for VSA expressed by CS2 PEs in plasma from NI (*n* = 3), MG HIV− (*n* = 23), and MG HIV+ donors (*n* = 23). Data shown are MFIs for IgG1 and IgG3 from individual donors gated on EtBr positive cells (PEs). Horizontal bars indicate the median. Inserts show contour plots of a NI, MG HIV−, and MG HIV+ donor, respectively. Statistical significance was assessed by Mann-Whitney rank sum test. *MG HIV− versus MG HIV+ *p* < 0.001. MG HIV−, HIV-negative MG; MG HIV+, HIV-infected MG; NI, nonimmune.

HIV infection has been reported to partially impair the production of VSA-PAM-specific IgG in pregnancy [18]. This observation suggests that HIV-infected women might have an opsonic phagocytic defect compared to their HIV-negative counterparts, resulting in an impaired ability to clear PAM PEs. We therefore compared the plasma opsonizing activity of HIV-negative and HIV-infected pregnant women. Tables S2 and S3 display the characteristics of the participants whose plasma samples were used in the following experiments; HIV-negative and HIV-infected pregnant women did not significantly differ on the basis of age, place of residence, placental malaria prevalence, or placental parasite density. As expected, plasma from HIV-negative and HIV-infected PG women had uniformly low plasma opsonizing activity (Figure 4A). Plasma from HIV-negative MG women, however, strongly induced the opsonic phagocytosis of CS2 PEs, while plasma from HIV-infected MG women had significantly lower plasma opsonizing activity (median phagocytic index 251 [IQR 93–397] versus 46 [IQR 18–195], respectively; *p* = 0.006 [U = 143.0], by Mann-Whitney rank sum test) (Figure 4B). Since IgG1 and IgG3 subclasses mediate the opsonic phagocytic uptake of CSA-binding PEs, we next tested whether lower levels of these IgG subclasses could explain the observed opsonic phagocytic defect in plasma from HIV-infected MG women. Levels of IgG subclasses specific for VSA expressed by CS2 PEs were measured in plasma from HIV-negative and HIV-infected MG women using flow cytometry. Median plasma levels of IgG1 and IgG3 with specificity for CS2 VSA were significantly lower in HIV-infected than in HIV-negative MG women (IgG1: MFI 13 [IQR 11–20] versus 30 [IQR 23–41], *p* < 0.001 (U = 111.0); IgG3: MFI 17 [IQR 14–23] versus 28 [IQR 23–37], *p* < 0.001 (U = 111.5), by Mann-Whitney rank sum test) (Figure 5).

### Correlation between Plasma Opsonizing Activity and Levels of VSA-PAM-Specific IgG

**Figure 6:**
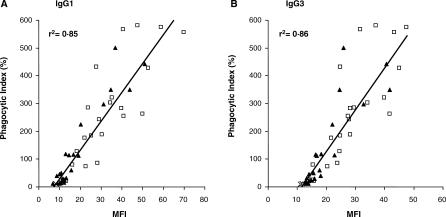
Correlation between Levels of VSA-PAM-Specific IgG1 and IgG3 and Plasma Opsonizing Activity Plasma levels of IgG1 and IgG3 subclasses specific for VSA expressed by CS2 PEs measured by flow cytometry were compared with their corresponding phagocytic index. “X” symbols represent plasma from nonimmune donors (*n* = 3), open squares represent plasma from multigravid HIV-negative women (*n* = 23), and solid triangles represent plasma from multigravid HIV-infected women (*n* = 23). Levels of IgG1 and IgG3 specific for VSA expressed by CS2 PEs correlated significantly with plasma opsonizing activity (*r* ^2^ = 0.85 and *r* ^2^ = 0.86 respectively, *p* < 0.001). Statistical significance was assessed by the Spearman correlation coefficient, with *F*-test.

Finally, we examined the relationship between plasma opsonizing activity and levels of VSA-PAM-specific total (Figure S4) and cytophilic IgG. There was a strong correlation between levels of both IgG1 and IgG3 subclasses specific for VSA expressed by CS2 PEs and opsonic phagocytosis (*r* ^2^ = 0.85 and *r* ^2^ = 0.86, respectively, *p* < 0.001 (*F* = 179.7, DFn = 1, DFd = 44 for IgG1; *F* = 122.5, DFn = 1, DFd = 44 for IgG3), by Spearman's correlation coefficient, with *F*-test) (Figure 6). Collectively these data demonstrate that many HIV-infected women have an opsonic phagocytic defect, which can be attributed to lower plasma levels of VSA-PAM-specific cytophilic antibodies.

## Discussion

In this study we demonstrate that CSA-binding PEs are cleared by opsonic phagocytosis in a sex-specific and parity-dependent manner, and that VSA-PAM-specific IgG1 and IgG3 mediate this response. In addition, we demonstrate that plasma from HIV-infected MG women had significantly lower opsonizing activity than HIV-negative MG women, and this defect was associated with lower levels of VSA-PAM-specific IgG1 and IgG3.

Protection against PAM is associated with plasma levels of VSA-PAM-specific IgG, which are generally low in PG women and nonpregnant individuals, and increase with increasing gravidity [16,17,21,29]. However, the exact mechanisms by which VSA-PAM-specific IgG protects against PAM and its clinical consequences remain incompletely understood. A number of studies have implicated adhesion blocking as a major mechanism by which VSA-PAM-specific IgG prevents the placental accumulation of CSA-binding PEs and protects against PAM [15,19,30]. However, other studies have found a lack of correlation between plasma antiadhesion activity, levels of VSA-specific IgG (measured by flow), and clinical protection against PAM, suggesting that other immune mechanisms may also be involved [18,20,21].

We evaluated the role of opsonic phagocytosis as an effector mechanism by which VSA-PAM-specific IgG might confer protection against PAM. Our results demonstrate that plasma from MG Kenyan women, but not from PG women or sympatric men, strongly promoted the opsonic phagocytosis of CSA-binding PEs (Figure 1). This sex-specific and parity-dependent pattern of plasma opsonizing activity parallels the production of VSA-PAM-specific IgG and clinical protection against PAM. Opsonic phagocytosis was found to depend on VSA-PAM-specific IgG1 and IgG3, since MG plasma depleted of these subclasses lacked opsonizing activity (Figure 3B). These data support the hypothesis that VSA-PAM-specific IgG promotes the opsonic phagocytic clearance of CSA-binding PEs and further, that VSA-PAM-specific IgG1 and IgG3 mediate this response. Our observations provide a new putative mechanism by which VSA-PAM-specific IgG may protect against PAM. These data suggest that in addition to adhesion blockade [6,15], opsonic immune mechanisms may also contribute to protection against PAM. Collectively, these observations extend the function of VSA-PAM-specific IgG to suggest that low levels of antibodies directed against CSA binding parasites in primigravidae [14,17,21,23,29] may increase their susceptibility to PAM in at least two ways: (1) through the inability to block adhesion of PEs to CSA in the placenta, and (2) through the inability to clear CSA-binding PEs by opsonic phagocytosis.

Our second aim was to provide a mechanistic explanation for the increased susceptibility of HIV-infected pregnant women to PAM. In a previous study, HIV-infected pregnant Malawian women were found to have lower plasma levels of total IgG with specificity for VSA expressed by CS2 PEs [18]. Plasma anti-adhesion activity was not associated with HIV status, suggesting that HIV infection may impair the production of other VSA-PAM-specific antibodies, for example opsonizing antibodies [18,21]. A major finding of our study is that plasma from HIV-infected MG women demonstrated significantly lower median opsonizing activity toward CS2 PEs than plasma from HIV-negative MG women (Figure 5B). This observed phagocytic defect could be explained by lower median levels of VSA-PAM-specific IgG1 and IgG3 in plasma from HIV-infected MG women (Figure 6). By contrast, plasma opsonizing activity of HIV-negative and HIV-infected PG women was not significantly different (Figure 5A). This is not surprising, since plasma from HIV-negative PG women already displayed very low opsonizing activity, which could not be further impaired by HIV infection.

Our findings that HIV-infected MG women have lower median plasma levels of VSA-PAM-specific IgG1 and IgG3, resulting in lower plasma opsonizing activity toward CSA-binding PEs, offer a novel mechanism by which HIV infection may increase the susceptibility of MG women to PAM. Lower plasma opsonizing activity suggests that HIV-infected MG women have an impaired ability to clear PAM PEs, which could help to explain why HIV-infected women experience more frequent and higher parasite densities than their HIV-negative counterparts [31]. Furthermore, studies have shown that the HIV-associated risk of PAM is consistently greater in multigravidae than in primigravidae [5]. Our data reflect this pattern of HIV-associated susceptibility to PAM since impairment of opsonizing activity and clearance of CSA-binding PEs by HIV infection was a unique property of plasma from MG and not PG women. Importantly, our study indicates that HIV infection may impair malaria-specific humoral immune responses early on during the course of infection, since many of the HIV-infected women tested had CD4^+^ T cell counts greater than 400 cells/μl.

Our findings have implications for the rational design and evaluation of vaccines to protect women against PAM. Although additional epidemiologic studies are required to confirm the association, our data implicate levels of VSA-PAM-specific IgG1 and IgG3 and opsonic phagocytosis of CSA-binding PEs as potentially important immune correlates of protection against PAM. This implication suggests that candidate PAM vaccines eliciting cytophilic VSA-PAM-specific IgG1 and IgG3 subclasses may confer increased protection against PAM.

Limitations of this study include the use of CS2, a CSA-binding laboratory isolate, and not placental isolates. Although CS2 expresses *var2csa* as do placental isolates, and although CS2 has an antigenic and adhesive phenotype similar to fresh placental isolates [13,18], our data require repetition with placental isolates to confirm the generalisability of our findings. Future epidemiological studies will also be required to confirm whether plasma opsonizing activity is a robust correlate of protective immunity against PAM.

In conclusion, we have identified the opsonic phagocytosis of CSA-binding parasites as a potentially important effector function by which VSA-PAM-specific cytophilic IgG may mediate protection against PAM. We have further shown that HIV-infected MG women have lower median plasma opsonizing activity toward CSA-binding PEs than their HIV-negative counterparts, reflecting lower plasma levels of cytophilic VSA-PAM-specific IgG subclasses. These data provide insight into the IgG-mediated mechanisms of protection against PAM and suggest a novel mechanism by which HIV may impair immunity to malaria in pregnancy.

## Supporting Information

Figure S1 Comparison of Phagocytosis of Plasma Opsonized CSA-Binding PEs between Human Monocytes and CD36^−/−^ Murine Macrophages Phagocytic index of CSA-binding PEs opsonized with plasma from non-immune (NI), malaria-exposed PG or malaria-exposed MG women by human monocytes (A) or *Cd36* ^−/−^ murine macrophages (B). The higher baseline phagocytosis observed with human monocytes is the result of scavenger receptor (e.g., CD36) mediated uptake. MG1, HIV-1 infected MG woman; MG2 to MG4, HIV-1 negative MG women; PG1, HIV-1 negative PG woman; PG2, HIV-1 infected PG woman. Data are means with SD of triplicates.
(40 KB PPT)

Figure S2 Plasma Opsonizing Activity of CD36-Binding Parasitized Erythrocytes Is Not Sex-Specific or Parity-Dependent (A) Box (median and IQR) and whisker (range) plot showing phagocytic index of CD36-binding (ITG) PEs opsonized with plasma from malaria-exposed males (M) (*n* = 12) or malaria-exposed MG women (*n* = 10).
(B) Box (median and IQR) and whisker (range) plot showing phagocytic index of CD36- binding (ITG) PEs opsonized with plasma from malaria-exposed PG (*n* = 15) or malaria-exposed MG (*n* = 5) donors. All donor plasma samples were assayed in duplicate and both replicates were included. Statistical significance was assessed by Mann-Whitney rank sum test. None of the comparisons were significant.
(12 KB PDF)

Figure S3 Plasma Heat Inactivation Does Not Affect Opsonizing Activity Phagocytosis of CSA-binding PEs opsonized with malaria-exposed MG plasma by *Cd36* ^−/−^ murine macrophages. Solid bars indicated non-heat inactivated plasma and hatched bars indicate heat-inactivated plasma. Data are means with SD of triplicates. Plasma was heat inactivated for 1 h at 56 °C prior to use.
(11 KB PDF)

Figure S4 Correlation between Levels of VSA-PAM-Specific Total IgG and Plasma Opsonizing Activity Plasma levels of total IgG specific for VSA expressed by CS2 PEs measured by flow cytometry were compared with their corresponding phagocytic index. Open squares represent plasma from HIV-negative MG women (*n* = 23), and solid triangles represent plasma from HIV-infected MG women (*n* = 23). Levels of total IgG specific for VSA expressed by CS2 PEs correlated significantly with plasma opsonizing activity (*r* ^2^ = 0.787, *p* < 0.001). Statistical significance was assessed by the Spearman's correlation coefficient.
(10 KB PDF)

Table S1 Characteristics of Participants (Used in Figure 1) According to Parity (12 KB PDF)

Table S2 Characteristics of Primigravid Participants (Used in Figure 4A) According to HIV Status (12 KB PDF)

Table S3 Characteristics of Multigravid Participants (Used in Figure 4B) According to HIV Status (12 KB PDF)

## Abbreviations

CSA: chondroitin sulfate A
EtBr: ethidium bromide
MFI: mean fluorescence intensity
MG: multigravid
PAM: pregnancy-associated malaria
PE: parasitized erythrocyte
PfEMP1: *Plasmodium falciparum* erythrocyte membrane 1
PG: primigravid
SD: standard deviation
VSA: variant surface antigens
