# Supplementary material for: HIV Impairs Opsonic Phagocytic Clearance of Pregnancy-Associated Malaria Parasites
Source: PLoS Med. 2007 May 29;4(5):e181. doi: 10.1371/journal.pmed.0040181 (PMC1880852; doi:10.1371/journal.pmed.0040181)
Supplement: Figure S1 — Phagocytic index of CSA-binding PEs opsonized with plasma from non-immune (NI), malaria-exposed PG or malaria-exposed MG women by human monocytes (A) or Cd36 −/− murine macrophages (B). The higher baseline phagocytosis observed with human monocytes is the result of scavenger receptor (e.g., CD36) mediated uptake. MG1, HIV-1 infected MG woman; MG2 to MG4, HIV-1 negative MG women; PG1, HIV-1 negative PG woman; PG2, HIV-1 infected PG woman. Data are means with SD of triplicates. (40 KB PPT) [file pmed.0040181.sg001.ppt]

## Slide 1
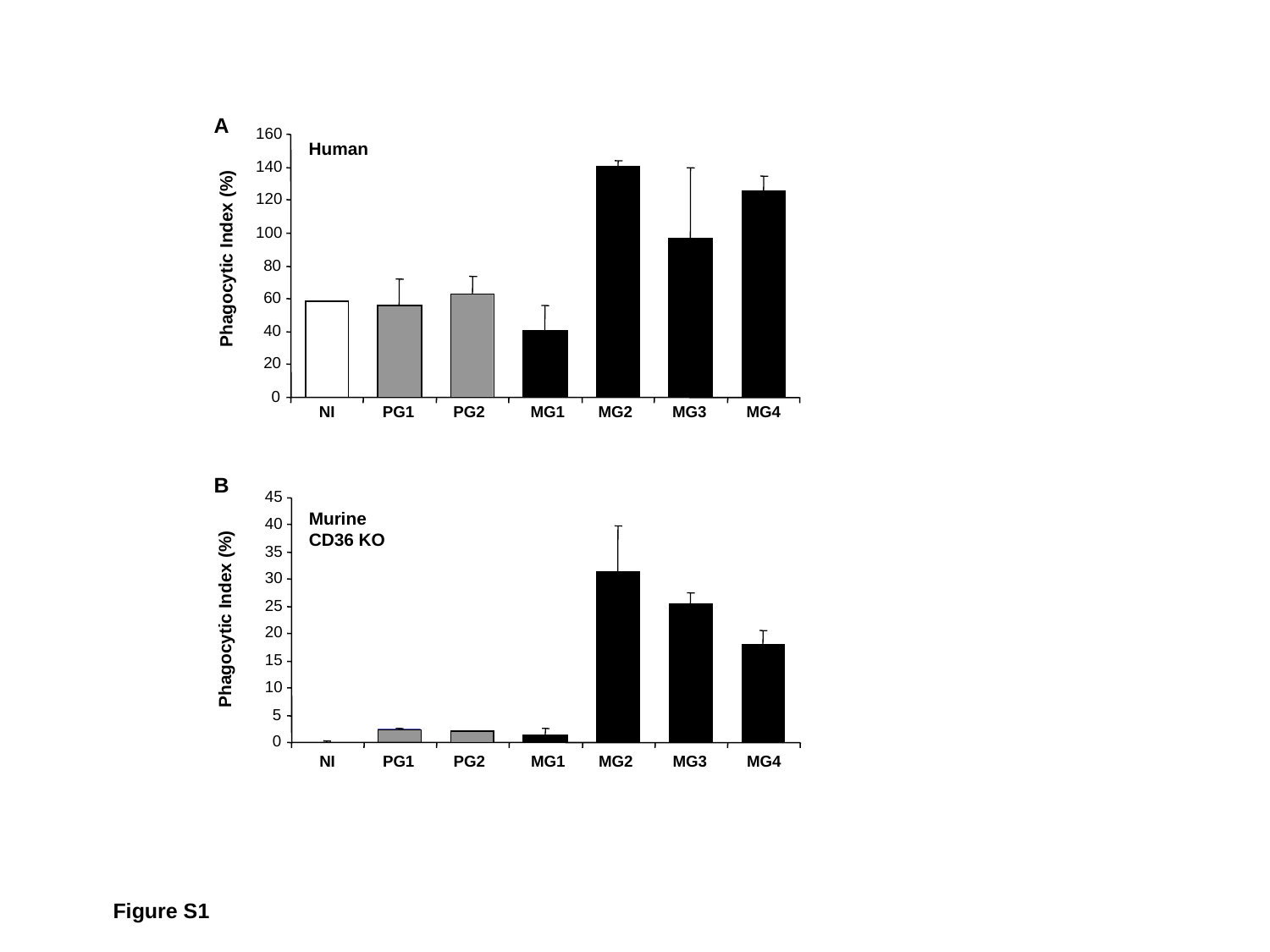

A
160
Human
140
120
100
Phagocytic Index (%)
80
60
40
20
0
NI
PG1
PG2
MG1
MG2
MG3
MG4
B
45
Murine
CD36 KO
40
35
30
25
Phagocytic Index (%)
20
15
10
5
0
NI
PG1
PG2
MG1
MG2
MG3
MG4
Figure S1
