# Supplementary figures and images for: HIV Impairs Opsonic Phagocytic Clearance of Pregnancy-Associated Malaria Parasites
Source: PLoS Med. 2007 May 29;4(5):e181. doi: 10.1371/journal.pmed.0040181 (PMC1880852; doi:10.1371/journal.pmed.0040181)

## Slide 1
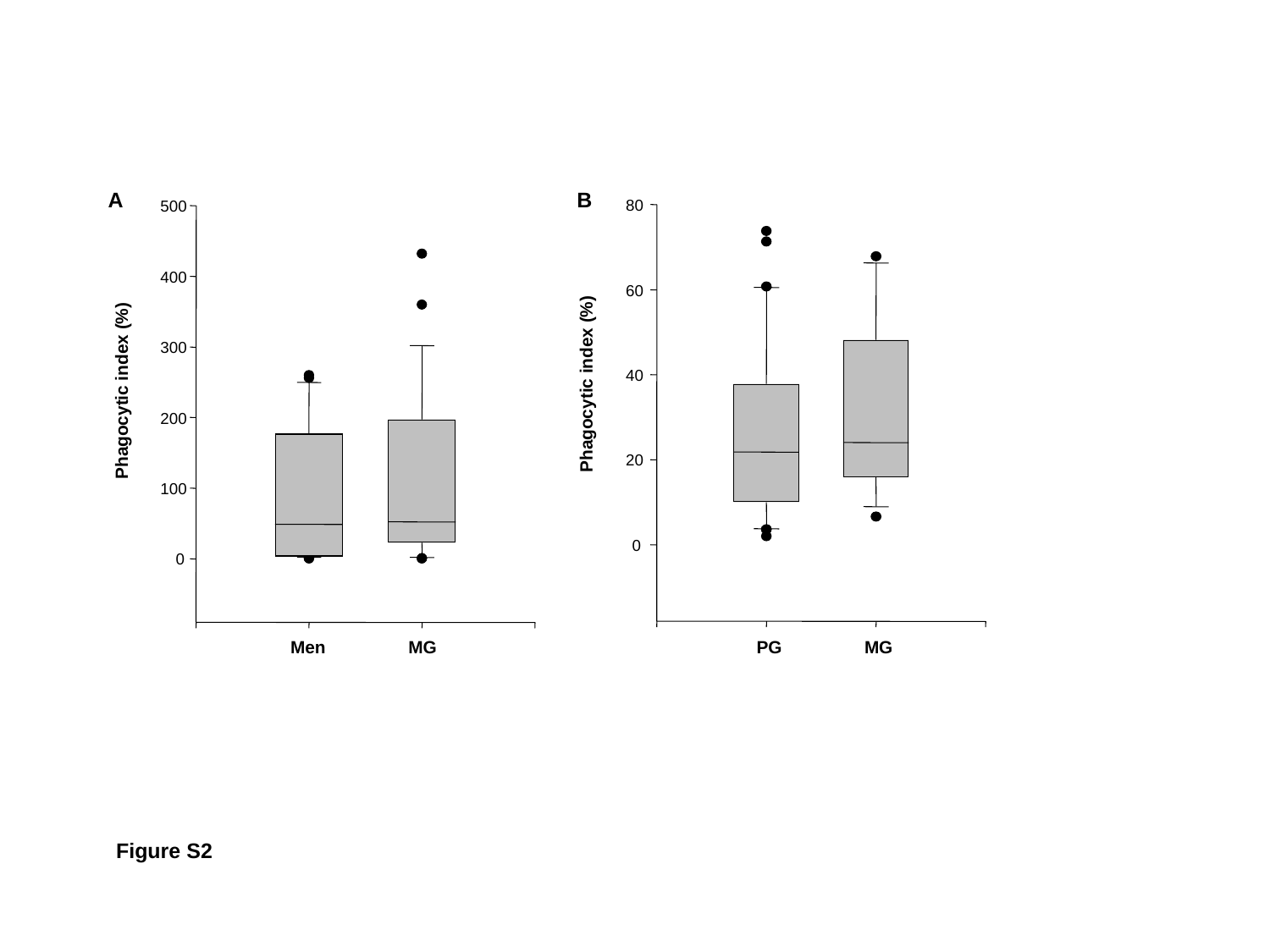

A
B
80
60
Phagocytic index (%)
40
20
0
 PG
 MG
500
400
300
Phagocytic index (%)
200
100
0
Men
MG
Figure S2

Supplement: Figure S2 — (A) Box (median and IQR) and whisker (range) plot showing phagocytic index of CD36-binding (ITG) PEs opsonized with plasma from malaria-exposed males (M) (n = 12) or malaria-exposed MG women (n = 10). (B) Box (median and IQR) and whisker (range) plot showing phagocytic index of CD36- binding (ITG) PEs opsonized with plasma from malaria-exposed PG (n = 15) or malaria-exposed MG (n = 5) donors. All donor plasma samples were assayed in duplicate and both replicates were included. Statistical significance was assessed by Mann-Whitney rank sum test. None of the comparisons were significant. (12 KB PDF) [file pmed.0040181.sg002.ppt]

## Slide 1
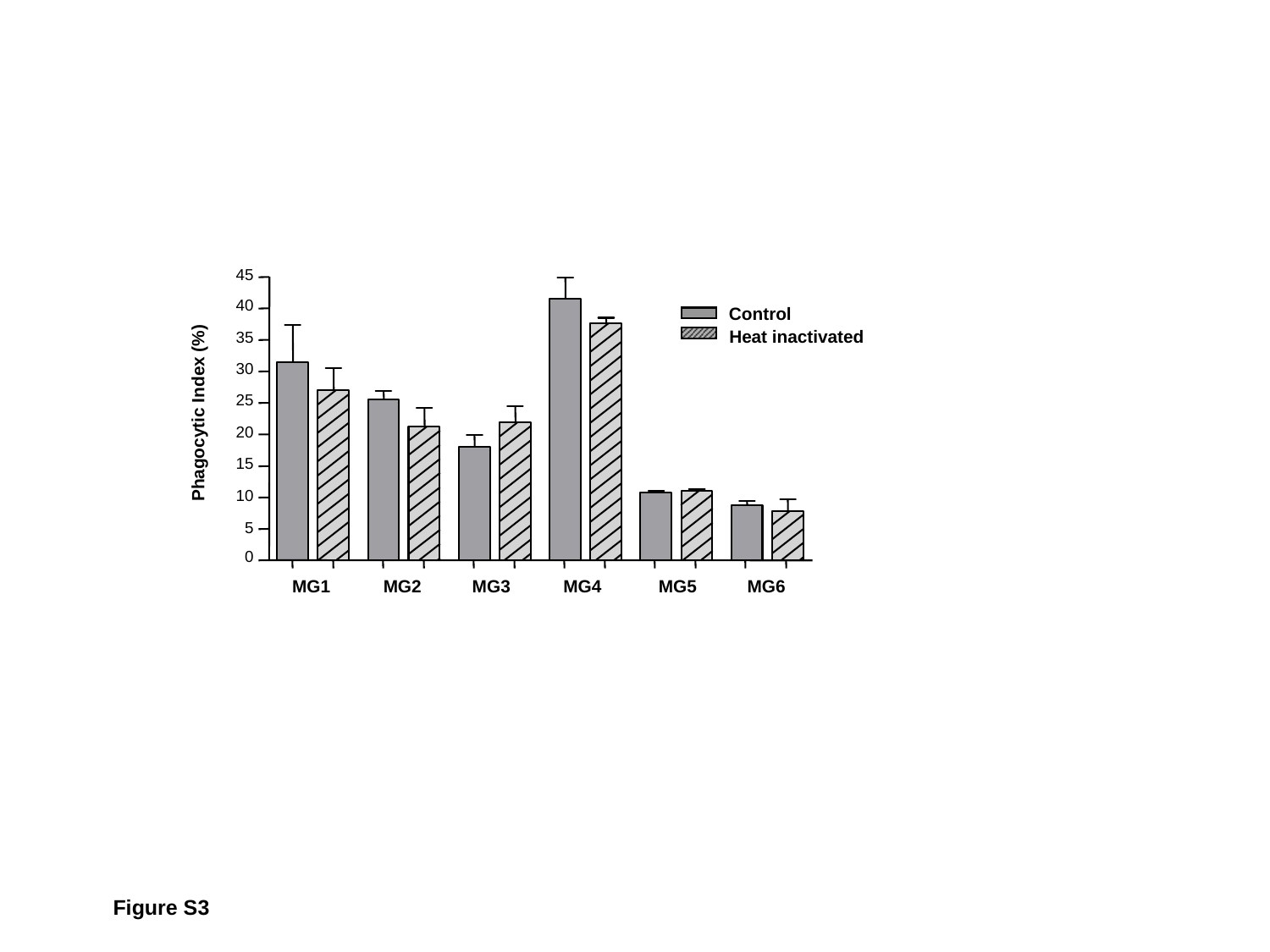

45
40
Control
Heat inactivated
35
30
25
Phagocytic Index (%)
20
15
10
5
0
MG1
MG2
MG3
MG4
MG5
MG6
Figure S3

Supplement: Figure S3 — Phagocytosis of CSA-binding PEs opsonized with malaria-exposed MG plasma by Cd36 −/− murine macrophages. Solid bars indicated non-heat inactivated plasma and hatched bars indicate heat-inactivated plasma. Data are means with SD of triplicates. Plasma was heat inactivated for 1 h at 56 °C prior to use. (11 KB PDF) [file pmed.0040181.sg003.ppt]

## Slide 1
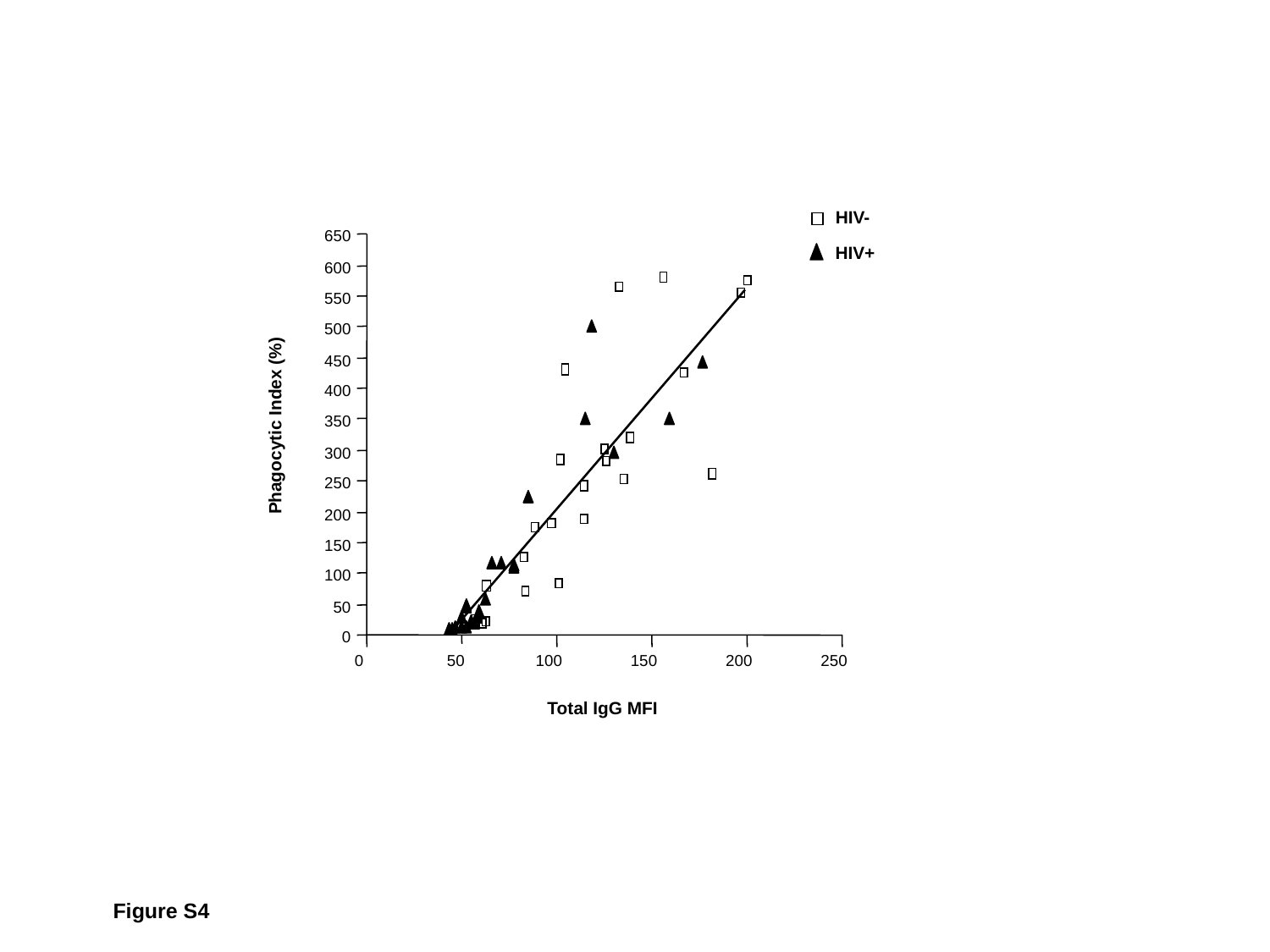

HIV-
650
HIV+
600
550
500
450
400
350
Phagocytic Index (%)
300
250
200
150
100
50
0
0
50
100
150
200
250
Total IgG MFI
Figure S4

Supplement: Figure S4 — Plasma levels of total IgG specific for VSA expressed by CS2 PEs measured by flow cytometry were compared with their corresponding phagocytic index. Open squares represent plasma from HIV-negative MG women (n = 23), and solid triangles represent plasma from HIV-infected MG women (n = 23). Levels of total IgG specific for VSA expressed by CS2 PEs correlated significantly with plasma opsonizing activity (r 2 = 0.787, p < 0.001). Statistical significance was assessed by the Spearman's correlation coefficient. (10 KB PDF) [file pmed.0040181.sg004.ppt]
