# Supplementary material for: HIV Impairs Opsonic Phagocytic Clearance of Pregnancy-Associated Malaria Parasites
Source: PLoS Med. 2007 May 29;4(5):e181. doi: 10.1371/journal.pmed.0040181 (PMC1880852; doi:10.1371/journal.pmed.0040181)
Supplement: Table S1 — (12 KB PDF) [file pmed.0040181.st001.doc]

**Supplemental Table 1**. Characteristics of participants (used in Figure 1) according to parity.

| **Variable** | Subcategory | All women **(n = 37)** | Primigravid **(n = 21)** | Multigravid **(n = 16)** | *P* value^a^ |
| --- | --- | --- | --- | --- | --- |
| Age^b^ |  | 21.7 (4.6) | 18.9 (1.9) | 25.4 (4.5) | <0.001 |
| Gravidity | 1 | 21 (56.8%) | 21 (100%) | NA |  |
|  | 3 | 10 (27.0%) | NA | 10 (62.5%) |  |
|  | >3 | 6 (16.2%) | NA | 6 (37.5%) |  |
| Place of residence | Urban | 26 (70.3%) | 15 (71.4%) | 11 (68.8%) | 1.0 |
|  | Semi-urban | 11 (29.7%) | 6 (28.6%) | 5 (31.2%) |  |
| Season of delivery | Wet | 23 (62.2%) | 13 (61.9%) | 10 (62.5%) | 1.0 |
|  | Dry | 14 (37.8%) | 8 (38.1%) | 6 (37.5%) |  |
| Placenta malaria | Negative | 25 (67.6%) | 15 (71.4%) | 10 (62.5%) | 0.73 |
|  | Positive | 12 (32.4%) | 6 (28.6%) | 6 (37.5%) |  |
| Placental parasitemia^c^ |  | 50.3 (5-951) | 41.8 (5-82) | 66.2 (12-951) | 0.91 |

^a^ All comparisons are between primgravid and multigravid. Statistical significance assessed by: unpaired Student’s t-test (for Age), Mann-Whitney (for Placental parasitemia), and Fisher’s exact (for all others).

^b^ Ages (in years) shown as means with SD.

^c^ Placental parasitemia (parasites/μl) reported for placenta malaria positive women only. Parasitemias shown as geometric means with range.
