# Supplementary material for: HIV Impairs Opsonic Phagocytic Clearance of Pregnancy-Associated Malaria Parasites
Source: PLoS Med. 2007 May 29;4(5):e181. doi: 10.1371/journal.pmed.0040181 (PMC1880852; doi:10.1371/journal.pmed.0040181)
Supplement: Table S2 — (12 KB PDF) [file pmed.0040181.st002.doc]

**Supplemental Table 2**. Characteristics of primigravid participants (used in Figure 4a) according to HIV status.

| **Variable** | Subcategory | All primigravid **(n = 20)** | HIV-negative **(n = 9)** | HIV-positive **(n = 11)** | *P* value^a^ |
| --- | --- | --- | --- | --- | --- |
| Age^b^ |  | 19.21 (2.6) | 18.8 (3.1) | 19.6 (2.2) | 0.52 |
| Gravidity | 1 | 20 (100%) | 9 (100%) | 11 (100%) |  |
|  | 3 | NA | NA | NA |  |
|  | >3 | NA | NA | NA |  |
| Place of residence | Urban | 16 (80.0%) | 7 (77.8%) | 9 (81.8%) | 1.0 |
|  | Semi-urban | 4 (20.0%) | 2 (22.2%) | 2 (18.2%) |  |
| Season of delivery | Wet | 9 (45.0%) | 2 (22.2%) | 7 (63.6%) | 0.092 |
|  | Dry | 11 (55.0%) | 7 (77.8%) | 4 (36.4%) |  |
| Placenta malaria | Negative | 11 (55.0%) | 5 (55.6%) | 6 (54.5%) | 1.0 |
|  | Positive | 9 (45.0%) | 4 (44.4%) | 5 (45.5%) |  |
| Placental parasitemia^c^ |  | 117.6 (3-1988) | 488.2 (73-1988) | 37.7 (3-1420) | 0.30 |

^a^ All comparisons are between HIV-negative and HIV-positive. Statistical significance assessed by: unpaired Student’s t-test (for Age), Mann-Whitney (for Parasitemia), and Fisher’s exact (for all others).

^b^ Ages (in years) shown as means with SD.

^c^ Placental parasitemia (parasites/μl) reported for placenta malaria positive women only. Parasitemias shown as geometric means with range.
