# Supplementary material for: HIV Impairs Opsonic Phagocytic Clearance of Pregnancy-Associated Malaria Parasites
Source: PLoS Med. 2007 May 29;4(5):e181. doi: 10.1371/journal.pmed.0040181 (PMC1880852; doi:10.1371/journal.pmed.0040181)
Supplement: Table S3 — (12 KB PDF) [file pmed.0040181.st003.doc]

**Supplemental Table 3**. Characteristics of multigravid participants (used in Figure 4b) according to HIV status.

| **Variable** | Subcategory | All multigravid **(n = 46)** | HIV-negative **(n = 23)** | HIV-positive **(n = 23)** | *P* value^a^ |
| --- | --- | --- | --- | --- | --- |
| Age^b^ |  | 26.2 (4.2) | 26.9 (4.7) | 25.5 (3.6) | 0.26 |
| Gravidity | 1 | NA | NA | NA | 0.76 |
|  | 3 | 25 (54.3%) | 12 (52.2%) | 13 (56.5%) |  |
|  | >3 | 21 (45.7%) | 11 (47.8%) | 10 (43.5%) |  |
| Place of residence | Urban | 39 (84.8%) | 17 (73.9%) | 22 (95.7%) | 0.096 |
|  | Semi-urban | 7 (15.2%) | 6 (26.1%) | 1 (4.3%) |  |
| Season of delivery | Wet | 24 (52.2%) | 11 (47.8%) | 13 (56.5%) | 0.77 |
|  | Dry | 22 (47.8%) | 12 (52.2%) | 10 (43.5%) |  |
| Placenta malaria | Negative | 40 (87.0%) | 18 (78.3%) | 22 (95.7%) | 0.19 |
|  | Positive | 6 (13.0%) | 5 (21.7%) | 1 (4.3%) |  |
| Placental parasitemia^c^ |  | 143.2 (3-1120) | 97.1 (3-1120) | 1001 (NA) | NA |

^a^ All comparisons are between HIV-negative and HIV-positive. Statistical significance assessed by: unpaired Student’s t-test (for Age), Mann-Whitney (for Parasitemia), and Fisher’s exact (for all others).

^b^ Ages (in years) shown as means with SD.

^c^ Placental parasitemia (parasites/μl) reported for placenta malaria positive women only. Parasitemias shown as geometric means with range.
